# Supplementary material for: A transcriptomic microglia taxonomy across mouse and human pathologies
Source: Nat Immunol. 2026 Mar 25;27(5):1066–80. doi: 10.1038/s41590-026-02472-z (PMC7618987; doi:10.1038/s41590-026-02472-z)
Supplement: Supplementary file 2 — Reporting Summary [file 41590_2026_2472_MOESM2_ESM.pdf]

nature portfolio

Corresponding author(s): Prof. Dr. Marco Prinz

Last updated by author(s): Feb 10, 2026

Reporting Summary

Nature Portfolio wishes to improve the reproducibility of the work that we publish. This form provides structure for consistency and transparency in reporting. For further information on Nature Portfolio policies, see our Editorial Policies and the Editorial Policy Checklist.

Statistics

For all statistical analyses, confirm that the following items are present in the figure legend, table legend, main text, or Methods section.

n/a	Confirmed	
☐	☒	The exact sample size (*n*) for each experimental group/condition, given as a discrete number and unit of measurement
☐	☒	A statement on whether measurements were taken from distinct samples or whether the same sample was measured repeatedly
☐	☒	The statistical test(s) used AND whether they are one- or two-sided<br>*Only common tests should be described solely by name; describe more complex techniques in the Methods section.*
☒	☐	A description of all covariates tested
☐	☒	A description of any assumptions or corrections, such as tests of normality and adjustment for multiple comparisons
☐	☒	A full description of the statistical parameters including central tendency (e.g. means) or other basic estimates (e.g. regression coefficient) AND variation (e.g. standard deviation) or associated estimates of uncertainty (e.g. confidence intervals)
☐	☒	For null hypothesis testing, the test statistic (e.g. *F*, *t*, *r*) with confidence intervals, effect sizes, degrees of freedom and *P* value noted<br>*Give P values as exact values whenever suitable.*
☒	☐	For Bayesian analysis, information on the choice of priors and Markov chain Monte Carlo settings
☒	☐	For hierarchical and complex designs, identification of the appropriate level for tests and full reporting of outcomes
☒	☐	Estimates of effect sizes (e.g. Cohen's *d*, Pearson's *r*), indicating how they were calculated

Our web collection on statistics for biologists contains articles on many of the points above.

Software and code

Policy information about availability of computer code

Data collection

Sequencing and fastq file generation:
NextSeq 550 Control Software (NCS) v4.0.1.41 for the Illumina NextSeq 550 instrument
bcl2fastq v2.20 software for conversion of .bcl files into fastq files
NextSeq 1000/2000 Control Software (NCS) v1.2.0.36376 for the Illumina NextSeq 1000/2000 instrument
DRAGEN v3.8.4 software for sequencing and conversion of .bcl files into fastq files

Data processing:
Processed data such as counts matrix were generated with cellranger-7.1.0 with the GENCODE human genome release 35 or mouse genome release 33 as reference genomes.
For alignment of Visium HD data spaceranger-3.0.0. was used with Visium Human Transcriptome Probe Set v2.0 and GRCh38 human reference genome

Microscopy:
Fluorescence imaging: Olympus BX-61 with a color camera (Olympus DP71)
Pannoramic 250 FLASH II (3DHISTECH) Digital Slide Scanner
BZ-9000 (Keyence)

Confocal imaging:
TCS SP8 X (Leica, LAS X 3.5.7.23225) microscope using a 20x 0.75 NA (HC PL APO CS2 20x/0.75 IMM) objective
Zeiss LSM800 microscope

Mass Cytometry:
Hyperion Imaging Mass Cytometry system (Fluidigm)

Data analysis

R environment:
R version 4.4.1 (2024-06-14)
Platform: x86_64-pc-linux-gnuRunning under: Ubuntu 22.04.5 LTSMatrix products: defaultBLAS: /usr/lib/x86_64-linux-gnu/openblas-pthread/libblas.so.3 LAPACK: /usr/lib/x86_64-linux-gnu/openblas-pthread/libopenblasp-r0.3.20.so; LAPACK version 3.10.0locale: [1] LC_CTYPE=en_US.UTF-8 LC_NUMERIC=C LC_TIME=de_DE.UTF-8 LC_COLLATE=en_US.UTF-8 LC_MONETARY=de_DE.UTF-8 [6] LC_MESSAGES=en_US.UTF-8 LC_PAPER=de_DE.UTF-8 LC_NAME=C LC_ADDRESS=C LC_TELEPHONE=C [11] LC_MEASUREMENT=de_DE.UTF-8 LC_IDENTIFICATION=C time zone: Europe/Berlintzcode source: system (glibc)attached base packages:[1] grid stats4 stats graphics grDevices utils datasets methods base other attached packages: [1] SoupX_1.6.2 magrittr_2.0.3 CellChat_2.1.2 DoubletFinder_2.0.4 reshape2_1.4.4 [6] arrow_18.1.0.1 scales_1.3.0 gridExtra_2.3 circlize_0.4.16 ineq_0.2-13 [11] igraph_2.0.3 UCell_2.8.0 ComplexHeatmap_2.21.1 data.table_1.16.4 Azimuth_0.5.0 [16] shinyBS_0.61.1 RColorBrewer_1.1-3 SeuratWrappers_0.3.5 SeuratData_0.2.2.9001 EnhancedVolcano_1.22.0 [21] ggrepel_0.9.6 monocle3_1.3.4 SingleCellExperiment_1.26.0 SummarizedExperiment_1.34.0 GenomicRanges_1.56.2 [26] GenomeInfoDb_1.40.1 IRanges_2.38.1 S4Vectors_0.42.1 MatrixGenerics_1.16.0 matrixStats_1.5.0 [31] Biobase_2.64.0 BiocGenerics_0.50.0 ggtree_3.12.0 assertthat_0.2.1 clustree_0.5.1 [36] ggraph_2.2.1 Matrix_1.6-5 viridis_0.6.5 viridisLite_0.4.2 lubridate_1.9.4 [41] forcats_1.0.0 stringr_1.5.1 purrr_1.0.2 readr_2.1.5 tibble_3.2.1 [46] tidyverse_2.0.0 harmony_1.2.3 Rcpp_1.0.14 patchwork_1.3.0 cowplot_1.1.3 [51] ggplot2_3.5.1 reticulate_1.40.0 pheatmap_1.0.12 tidyr_1.3.1 dplyr_1.1.4 [56] Seurat_5.2.1 SeuratObject_5.0.2 sp_2.2-0
loaded via a namespace (and not attached): [1] ica_1.0-3 plotly_4.10.4 Formula_1.2-5 zlibbioc_1.50.0 [5] tidyselect_1.2.1 bit_4.5.0.1 doParallel_1.0.17 clue_0.3-66 [9] lattice_0.22-5 rjson_0.2.23 blob_1.2.4 rngtools_1.5.2 [13] S4Arrays_1.4.1 parallel_4.4.1 seqLogo_1.70.0 png_0.1-8 [17] cli_3.6.3 ggplotify_0.1.2 registry_0.5-1 ProtGenerics_1.36.0 [21] goftest_1.2-3 gargle_1.5.2 BiocIO_1.14.0 BiocNeighbors_1.22.0 [25] ggnetwork_0.5.13 Signac_1.14.0 uwot_0.2.2 curl_6.2.0 [29] mime_0.12 tidytree_0.4.6 stringi_1.8.4 backports_1.5.0 [33] XML_3.99-0.18 httpuv_1.6.15 AnnotationDbi_1.66.0 rappdirs_0.3.3 [37] splines_4.4.1 RcppRoll_0.3.1 DT_0.33 sctransform_0.4.1 [41] DBI_1.2.3 terra_1.8-21 jquerylib_0.1.4 withr_3.0.2 [45] systemfonts_1.2.1 reformulas_0.4.0 lmtest_0.9-40 tidygraph_1.3.1 [49] rtracklayer_1.64.0 BiocManager_1.30.25 htmlwidgets_1.6.4 fs_1.6.5 [53] biomaRt_2.60.1 statnet.common_4.11.0 SparseArray_1.4.8 cellranger_1.1.0 [57] annotate_1.82.0 zoo_1.8-12 JASPAR2020_0.99.10 XVector_0.44.0 [61] network_1.19.0 TFBSTools_1.42.0 UCSC.utils_1.0.0 TFMPvalue_0.0.9 [65] timechange_0.3.0 foreach_1.5.2 caTools_1.18.3 rhdf5_2.48.0 [69] pwalign_1.0.0 R.oo_1.27.0 poweRlaw_1.0.0 RSpectra_0.16-2 [73] irlba_2.3.5.1 fastDummies_1.7.5 gridGraphics_0.5-1 lazyeval_0.2.2 [77] yaml_2.3.10 survival_3.7-0 scattermore_1.2 crayon_1.5.3 [81] RcppAnnoy_0.0.22 progressr_0.15.1 tweenr_2.0.3 later_1.4.1 [85] ggridges_0.5.6 codetools_0.2-19 GlobalOptions_0.1.2 KEGGREST_1.44.1 [89] Rtsne_0.17 shape_1.4.6.1 Rsamtools_2.20.0 filelock_1.0.3 [93] pkgconfig_2.0.3 xml2_1.3.6 spatstat.univar_3.1-1 ggpubr_0.6.0 [97] GenomicAlignments_1.40.0 aplot_0.2.4 spatstat.sparse_3.1-0 BSgenome_1.72.0 [101] ape_5.8-1 gridBase_0.4-7 xtable_1.8-4 car_3.1-3 [105] plyr_1.8.9 httr_1.4.7 rbibutils_2.3 tools_4.4.1 [109] globals_0.16.3 broom_1.0.7 nlme_3.1-165 dbplyr_2.5.0 [113] hdf5r_1.3.10 shinyjs_2.1.0 lme4_1.1-36 digest_0.6.37 [117] farver_2.1.2 tzdb_0.4.0 AnnotationFilter_1.28.0 yulab.utils_0.2.0 [121] DirichletMultinomial_1.46.0 glue_1.8.0 cachem_1.1.0 BiocFileCache_2.12.0 [125] polyclip_1.10-7 generics_0.1.3 Biostrings_2.72.1 ggalluvial_0.12.5 [129] googledrive_2.1.1 presto_1.0.0 parallelly_1.42.0 RcppHNSW_0.6.0 [133] carData_3.0-5 minqa_1.2.8 pbapply_1.7-2 httr2_1.1.0 [137] spam_2.11-1 graphlayouts_1.2.2 gtools_3.9.5 ggsignif_0.6.4 [141] shiny_1.10.0 GenomeInfoDbData_1.2.12 R.utils_2.12.3 rhdf5filters_1.16.0 [145] RCurl_1.98-1.16 memoise_2.0.1 R.methodsS3_1.8.2 googlesheets4_1.1.1 [149] svglite_2.1.3 future_1.34.0 RANN_2.6.2 Cairo_1.6-2 [153] spatstat.data_3.1-4 rstudioapi_0.17.1 cluster_2.1.6 spatstat.utils_3.1-2 [157] hms_1.1.3 fitdistrplus_1.2-2 munsell_0.5.1 colorspace_2.1-1 [161] FNN_1.1.4.1 rlang_1.1.5 dotCall64_1.2 shinydashboard_0.7.2 [165] ggforce_0.4.2 coda_0.19-4 sna_2.8 CNEr_1.40.0 [169] remotes_2.5.0 iterators_1.0.14 abind_1.4-8 EnsDb.Hsapiens.v86_2.99.0 [173] treeio_1.28.0 Rhdf5lib_1.26.0 bitops_1.0-9 Rdpack_2.6.2 [177] promises_1.3.2 RSQLite_2.3.9 DelayedArray_0.30.1 GO.db_3.19.1 [181] compiler_4.4.1 prettyunits_1.2.0 boot_1.3-30 listenv_0.9.1 [185] BSgenome.Hsapiens.UCSC.hg38_1.4.5 tensor_1.5 MASS_7.3-61 progress_1.2.3 [189] BiocParallel_1.38.0 spatstat.random_3.3-2 R6_2.5.1 fastmap_1.2.0 [193] fastmatch_1.1-6 rstatix_0.7.2 ensembldb_2.28.1 ROCR_1.0-11 [197] SeuratDisk_0.0.0.9021 rsvd_1.0.5 gtable_0.3.6 KernSmooth_2.23-24 [201] miniUI_0.1.1.1 deldir_2.0-4 htmltools_0.5.8.1 bit64_4.6.0-1 [205] spatstat.explore_3.3-4 lifecycle_1.0.4 nloptr_2.1.1 restfulr_0.0.15 [209] sass_0.4.9 vctrs_0.6.5 spatstat.geom_3.3-5 NMF_0.28 [213] ggfun_0.1.8 future.apply_1.11.3 bslib_0.9.0 pillar_1.10.1 [217] GenomicFeatures_1.56.0 magick_2.8.5 jsonlite_1.8.9 GetoptLong_1.0.5

Python environment:
Package,VersionBabel,2.15.0Brotli,1.1.0GDAL,3.9.0HeapDict,1.0.1Markdown,3.6MarkupSafe,2.1.5PIMS,0.6.1Pint,0.24PyOpenGL,3.1.7PyQt5,5.15.9PyQt5-sip,12.12.2PySocks,1.7.1PyWavelets,1.4.1PyYAML,6.0.1QtPy,2.4.1Rtree,1.2.0absl-py,2.1.0aiobotocore,2.5.4aiohttp,3.9.5aioitertools,0.11.0aiosignal,1.3.1alabaster,0.7.16anndata,0.10.7annotated-types,0.7.0app-model,0.2.7appdirs,1.4.4array-api-compat,1.7.1asciitree,0.3.3asttokens,2.4.1astunparse,1.6.3attrs,23.2.0bin2cell,0.1.1bokeh,3.4.1botocore,1.31.17branca,0.7.2build,1.2.1cached-property,1.5.2cachey,0.2.1certifi,2024.6.2charset-normalizer,3.3.2click,8.1.7click-plugins,1.1.1cligj,0.7.2cloudpickle,3.0.0colorama,0.4.6colorcet,3.1.0comm,0.2.2contourpy,1.2.1cramjam,2.8.3csbdeep,0.8.0cycler,0.12.1cyto

olz,0.12.3dask,2024.2.1dask-expr,1.1.2dask-image,2023.8.1datashader,0.16.2debugpy,1.8.1decorator,5.1.1distributed,2024.2.1docrep,0.3.2docstring-parser,0.16docutils,0.21.2exceptiongroup,1.2.0executing,2.0.1fasteners,0.17.3fastparquet,2024.5.0fcsparser,0.2.8filelock,3.15.3fiona,1.9.6flatbuffers,24.3.25flexcache,0.3flexparser,0.3.1folium,0.16.0fonttools,4.53.0freetype-py,2.4.0frozenlist,1.4.1fsspec,2023.6.0gast,0.5.4geopandas,0.14.4get-annotations,0.1.2google-pasta,0.2.0grpcio,1.62.2h5py,3.11.0hsluv,5.0.4idna,3.7igraph,0.11.5imagecodecs,2024.6.1imageio,2.34.1imagesize,1.4.1importlib-metadata,7.1.0in-n-out,0.2.1inflect,7.2.1ipykernel,6.29.4ipython,8.25.0jedi,0.19.1jinja2,3.1.4jmespath,1.0.1joblib,1.4.2jsonschema,4.22.0jsonschema-specifications,2023.12.1jupyter-client,8.6.2jupyter-core,5.7.2keras,3.3.3kiwisolver,1.4.5lamin-utils,0.13.2lazy-loader,0.4legacy-api-wrap,1.4leidenalg,0.10.2llvmlite,0.42.0locket,1.0.0loguru,0.7.2louvain,0.8.2lz4,4.3.3magicgui,0.8.3mapclassify,2.6.1markdown-it-py,3.0.0matplotlib,3.8.4matplotlib-inline,0.1.7matplotlib-scalebar,0.8.1mdurl,0.1.2ml-dtypes,0.3.2more-itertools,10.3.0mpmath,1.3.0msgpack,1.0.8multidict,6.0.5multipledispatch,1.0.0multiscale-spatial-image,0.11.2munkres,1.1.4namex,0.0.8napari,0.4.19.post1napari-console,0.0.9napari-matplotlib,2.0.1napari-plugin-engine,0.2.0napari-spatialdata,0.4.1napari-svg,0.1.10natsort,8.4.0nest-asyncio,1.6.0networkx,3.3npe2,0.7.5numba,0.59.1numcodecs,0.12.1numpy,1.26.4numpydoc,1.7.0nvidia-cublas-cu12,12.1.3.1nvidia-cuda-cupti-cu12,12.1.105nvidia-cuda-nvrtc-cu12,12.1.105nvidia-cuda-runtime-cu12,12.1.105nvidia-cudnn-cu12,8.9.2.26nvidia-cufft-cu12,11.0.2.54nvidia-curand-cu12,10.3.2.106nvidia-cusolver-cu12,11.4.5.107nvidia-cusparse-cu12,12.1.0.106nvidia-nccl-cu12,2.20.5nvidia-nvjitlink-cu12,12.5.40nvidia-nvtx-cu12,12.1.105ome-zarr,0.9.0omnipath,1.0.8opencv-python,4.10.0.84opt-einsum,3.3.0optree,0.11.0packaging,24.1pandas,2.2.2param,2.1.0parso,0.8.4partd,1.4.2patsy,0.5.6pexpect,4.9.0pillow,10.3.0pip,24.0platformdirs,4.2.2ply,3.11pooch,1.8.2prompt-toolkit,3.0.47protobuf,4.25.3psutil,5.9.8psygnal,0.11.1ptyprocess,0.7.0pure-eval,0.2.2pyarrow,16.1.0pyarrow-hotfix,0.6pyconify,0.1.6pyct,0.5.0pydantic,2.7.4pydantic-compat,0.1.2pydantic-core,2.18.4pygeos,0.14pygments,2.18.0pynndescent,0.5.12pyparsing,3.1.2pyproj,3.6.1pyproject-hooks,1.1.0python-dateutil,2.9.0pytz,2024.1pyzmq,26.0.3qtconsole,5.5.2readfcs,1.1.8referencing,0.35.1requests,2.32.3rich,13.7.1rpds-py,0.18.1s3fs,2023.6.0scanpy,1.10.1scikit-image,0.23.2scikit-learn,1.5.0scipy,1.13.1seaborn,0.13.2session-info,1.0.0setuptools,70.0.0shapely,2.0.4shellingham,1.5.4sip,6.7.12six,1.16.0slicerator,1.1.0snowballstemmer,2.2.0sortedcontainers,2.4.0spatial-image,0.3.0spatialdata,0.1.2spatialdata-io,0.1.2spatialdata-plot,0.2.2sphinx,7.3.7sphinxcontrib-applehelp,1.0.8sphinxcontrib-devhelp,1.0.6sphinxcontrib-htmlhelp,2.0.5sphinxcontrib-jsmath,1.0.1sphinxcontrib-qthelp,1.0.7sphinxcontrib-serializinghtml,1.1.10squidpy,1.2.2stack-data,0.6.3stardist,0.9.1statsmodels,0.14.2stdlib-list,0.10.0superqt,0.6.7sympy,1.12.1tabulate,0.9.0tblib,3.0.0tensorboard,2.16.2tensorboard-data-server,0.7.0tensorflow,2.16.1tensorflow-estimator,2.15.0termcolor,2.4.0texttable,1.7.0threadpoolctl,3.5.0tifffile,2024.5.22tinycss2,1.3.0toml,0.10.2tomli,2.0.1tomli-w,1.0.0toolz,0.12.1torch,2.3.1tornado,6.4.1tqdm,4.66.4traitlets,5.14.3triton,2.3.1typeguard,4.3.0typer,0.12.3typing-extensions,4.12.2tzdata,2024.1umap-learn,0.5.5urllib3,1.26.19validators,0.28.3vispy,0.14.3wcwidth,0.2.13webencodings,0.5.1werkzeug,3.0.3wheel,0.43.0wrapt,1.16.0xarray,2024.6.0xarray-dataclasses,1.8.0xarray-datatree,0.0.14xarray-schema,0.0.3xarray-spatial,0.4.0xyzservices,2024.6.0yarl,1.9.4zarr,2.18.2zict,3.0.0zipp,3.19.2

For manuscripts utilizing custom algorithms or software that are central to the research but not yet described in published literature, software must be made available to editors and reviewers. We strongly encourage code deposition in a community repository (e.g. GitHub). See the Nature Portfolio guidelines for submitting code & software for further information.

Data

Policy information about availability of data

All manuscripts must include a data availability statement. This statement should provide the following information, where applicable:

- Accession codes, unique identifiers, or web links for publicly available datasets
- A description of any restrictions on data availability
- For clinical datasets or third party data, please ensure that the statement adheres to our policy

All sequencing data and processed seurat objects are publicly available. Human raw sequencing data are deposited at the European Genome–Phenome Archive (EGA; accession EGAS50000001289; https://ega-archive.org/studies/EGAS50000001289). Mouse raw sequencing data are available at the NCBI Gene Expression Omnibus (GEO; accession GSE304010; https://www.ncbi.nlm.nih.gov/geo/query/acc.cgi?&acc=GSE304010). Processed Seurat objects and the code to reproduce the figures are deposited at zenodo (DOI: 10.5281/zenodo.16938034; https://zenodo.org/uploads/16938034).

Research involving human participants, their data, or biological material

Policy information about studies with human participants or human data. See also policy information about sex, gender (identity/presentation), and sexual orientation and race, ethnicity and racism.

Reporting on sex and gender	Sex and gender were not disaggregated in the present study. Since the presented data is on cell identity and states it is expected to apply regardless of sex and therefore both sexes were included in the study. Individual datasets, except for lung brain metastasis, mostly consisted of cells derived from multiple participants and both sexes. We integrated the data for sex to minize the impact of sex on clustering. Details for integration are provided in methods section.
Reporting on race, ethnicity, or other socially relevant groupings	Limited information was available of the anonymized patients.
Population characteristics	The age range for samples was between the prenatal to 96 years. The tissue from control patients were at least 2 cm from the pathological focus and radiologically and histologically normal.
Recruitment	Participants were not prospectively recruited for this study. Human brain tissue samples were obtained either obtained during surgery or retrospectively from established brain biobanks, which collect post-mortem donations with prior consent from donors or their families. Case inclusion was therefore determined by tissue availability and existing clinical/pathological

	metadata rather than experimental recruitment criteria.<br>This approach introduces potential selection biases inherent to biobank-based studies. Donors may not be fully representative of the general population, as brain donation is influenced by factors such as disease severity, healthcare access, socioeconomic background, and willingness to participate in research. Additionally, variability in post-mortem interval, tissue preservation, and clinical documentation may introduce technical and metadata-related heterogeneity. These factors may limit generalizability but are unlikely to systematically bias within-cohort molecular comparisons, as all samples were processed using standardized protocols and analyzed under consistent experimental and computational workflows.
Ethics oversight	The study protocol was approved by the Ethics committee of the University of Freiburg Medical Center and local ethics committees.

Note that full information on the approval of the study protocol must also be provided in the manuscript.

Field-specific reporting

Please select the one below that is the best fit for your research. If you are not sure, read the appropriate sections before making your selection.

☒ Life sciences ☐ Behavioural & social sciences ☐ Ecological, evolutionary & environmental sciences

For a reference copy of the document with all sections, see nature.com/documents/nr-reporting-summary-flat.pdf

Life sciences study design

All studies must disclose on these points even when the disclosure is negative.

Sample size	The samples were acquired prospectively. No calculation of the sample size was performed. The sample size is in line with previous peer-reviewed studies for individual diseases (e.g. Movahedi et al, Nat Neurosci 2021; Friebel et al, Cell 2021; Klemm et al Cell 2021; Sankowski et al, Nat Neurosci 2019, Masuda et al. 2019).
Data exclusions	To minimize technical artifacts cells with unusual gene expression profiles were excluded. We also employed cut offs for number of transcripts and genes detected per cell across all the single cell and spatial transcriptomics datasets which are similar to previously published studies and comprehensively described in methods section.
Replication	We have included more than 3 patients per disease when available. Similarly for mouse samples we have harvested tissues from at least 3 animals. Additionally, the snRNA-seq data were validated by several orthogonal methods for example at protein levels using mass cytometry and immunohistochemistry as well as RNAscope based on smFISH with multiple biological replicates. We also validated the presence of several microglial transcriptional states in situ in patient samples via Nanostring CosMx and and Visium HD based single cell spatial transcriptomics.
Randomization	Randomization was not performed as the study does not include an intervention. Samples were collected prospectively and stratified according to the radiological and histological appearance as normal or pathological.
Blinding	Microscopy analyses were conducted by blinded experimenters. scRNA-Seq and mass cytometry data were analyzed by algorithms in an unsupervised manner. Due to the unsupervised manner of the analysis, blinding is not expected to affect the results.

Reporting for specific materials, systems and methods

We require information from authors about some types of materials, experimental systems and methods used in many studies. Here, indicate whether each material, system or method listed is relevant to your study. If you are not sure if a list item applies to your research, read the appropriate section before selecting a response.

Materials & experimental systems

n/a	Involved in the study	
☐	☒	Antibodies
☒	☐	Eukaryotic cell lines
☒	☐	Palaeontology and archaeology
☐	☒	Animals and other organisms
☒	☐	Clinical data
☒	☐	Dual use research of concern
☒	☐	Plants

Methods

n/a	Involved in the study	
☒	☐	ChIP-seq
☐	☒	Flow cytometry
☒	☐	MRI-based neuroimaging

Antibodies

Antibodies used	Anti-Olig2 Antibody, clone 211F1.1, Alexa Fluor®488 Conjugate Merck: Cat no. MABN50A4<br>RBFOX3/NeuN Antibody, clone 1B7, Alexa Fluor® 647 Conjugate Novus biologicals: Cat no. NBP1-92693AF647<br>Recombinant Anti-TMEM119 antibody clone 28-3 Abcam: Cat no. ab209064

	Alexa Fluor® 647 anti-mouse CD74 (CLIP) Antibody clone In1/CD74 Biolegend: Cat no. 151004<br><br>FKBP5 Polyclonal antibody Proteintech Cat. No. 14155-1-AP<br>Anti-P2RY12 antibody Sigma-Aldrich Cat. No. HPA014518<br>Human Osteoactivin/GPNMB Antibody RD Systems Cat. No. AF2550<br><br>Secondary antibodies<br>anti-rabbit Alexa Flour® 488, Thermofisher scientific Cat no: A-21206<br>Rabbit Anti-Goat Immunoglobulins/Biotin DAKO Cat No. E0466<br>donkey anti-rabbit-AF647 Life Technologies Cat. No. A31573
Validation	Immunohistochemistry antibodies were validated using positive and negative control stainings following the manufacturer's instructions. The antibodies were validated at different dilutions and pretreatments by experienced staff at the histology lab of the department. FACS antibodies were validated according to the manufacturer's instructions using all relevant isotype controls and fluorescence minus one controls.

Animals and other research organisms

Policy information about studies involving animals; ARRIVE guidelines recommended for reporting animal research, and Sex and Gender in Research

Laboratory animals	C57BL/6 WT mice or transgenic mice on C57BL/6 background were used for experiments. For experiments with transgenic animals, either WT littermates or cre negative litter mates were used. Depending on the model mice at different ages were used, the details for which are provided in the Supplemental table 1 of the manuscript.
Wild animals	Study did not invlove wild animals.
Reporting on sex	Only female mice were used in experiments.
Field-collected samples	Study did not involve samples collected from the field.
Ethics oversight	All animal experiments were approved and performed in accordance with national and institutional regulations (Regierungspräsidium Freiburg, approval numbers X17/01A, X-20/01A, G-19/084, G-18/044, G-19/124, G-20/49, G-22/094, G20/131, G-17/063; LAVES approval numbers 17-2697, 16/2338; Landesamt für Gesundheit und Soziales, Berlin, Registration numbers: G0312/16 and G0167/20, LAGeSO approval number G0031/21).

Note that full information on the approval of the study protocol must also be provided in the manuscript.

Plants

Seed stocks	Not applicable
Novel plant genotypes	Not applicable
Authentication	Not applicable

Flow Cytometry

Plots

Confirm that:

- ☐ The axis labels state the marker and fluorochrome used (e.g. CD4-FITC).
- ☐ The axis scales are clearly visible. Include numbers along axes only for bottom left plot of group (a 'group' is an analysis of identical markers).
- ☐ All plots are contour plots with outliers or pseudocolor plots.
- ☐ A numerical value for number of cells or percentage (with statistics) is provided.

Methodology

Sample preparation	Single-nucleus suspensions were prepared using the Frankenstein community protocol (https://www.protocols.io/view/frankenstein-protocol-for-nuclei-isolation-from-f-5jyl8nx98l2w/v2 accessed on July 1st 2022).

Instrument	Becton Dickinson FACSAria III, 17 color (Lasers: 375/405 nm, 488 nm, 561 nm, 633 nm)
Software	*Describe the software used to collect and analyze the flow cytometry data. For custom code that has been deposited into a community repository, provide accession details.*
Cell population abundance	*Describe the abundance of the relevant cell populations within post-sort fractions, providing details on the purity of the samples and how it was determined.*
Gating strategy	For single-nucleus mRNA sequencing DAPI+NeuN-Olig2- except for lung brain metastasis samples where all DAPI+ nuclei were sorted. The sorting data were not used for any inferences.

☐ Tick this box to confirm that a figure exemplifying the gating strategy is provided in the Supplementary Information.
