# Supplementary material for: A transcriptomic microglia taxonomy across mouse and human pathologies
Source: Nat Immunol. Author manuscript; Available in PMC 2026 Apr 9. (PMC7618987; doi:10.1038/s41590-026-02472-z)
Supplement: Supplementary file 1 [file EMS213289-supplement-Supplementary.pdf]

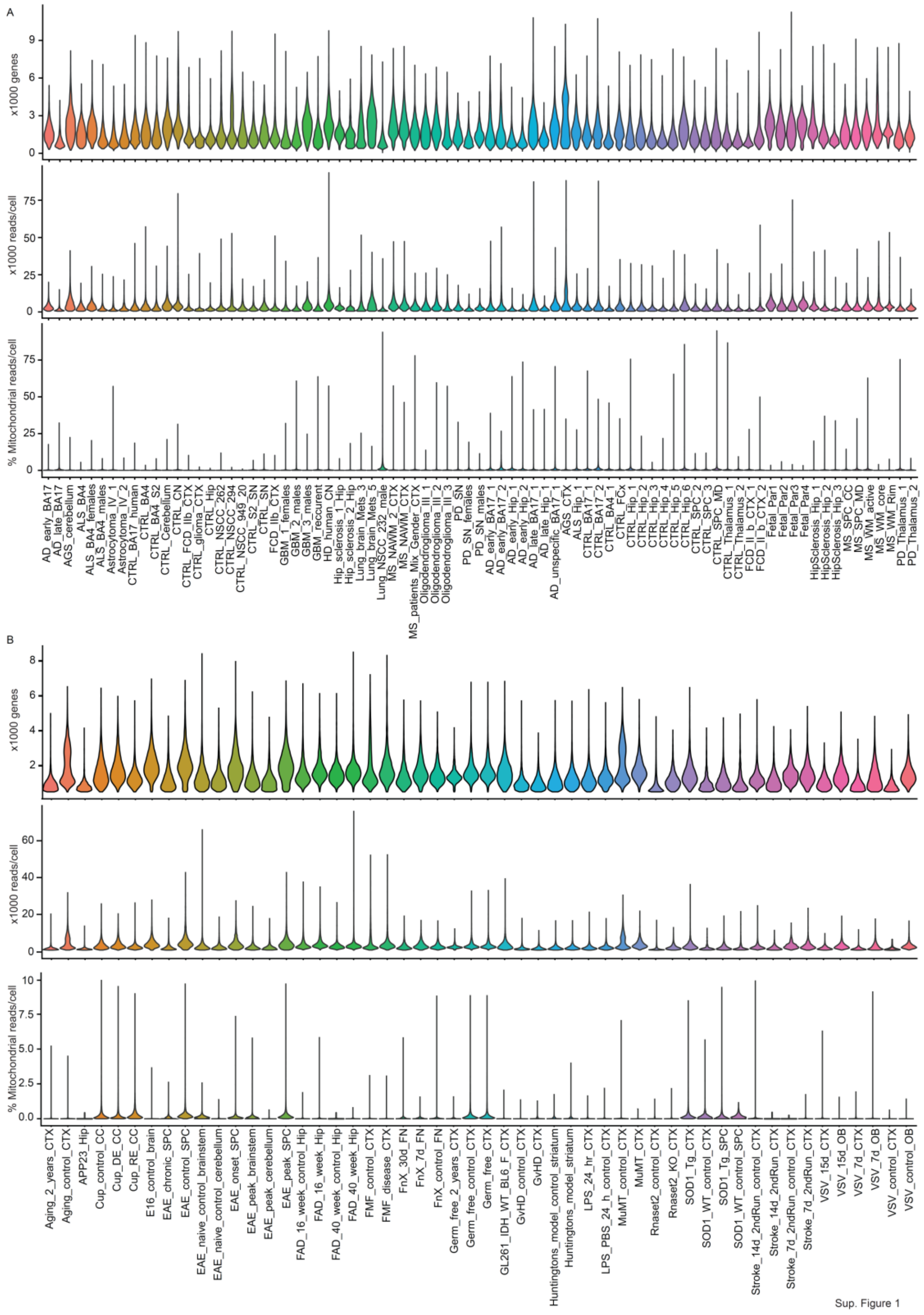

Supplementary Data Figure 1: Quality control metrics for human and mouse single nucleus transcriptomics libraries.

(A-B) Violin plots showing numbers of genes, reads per cell, and percentage of mitochondrial reads per cell in human (A) and mouse (B) 10x transcriptomics libraries.

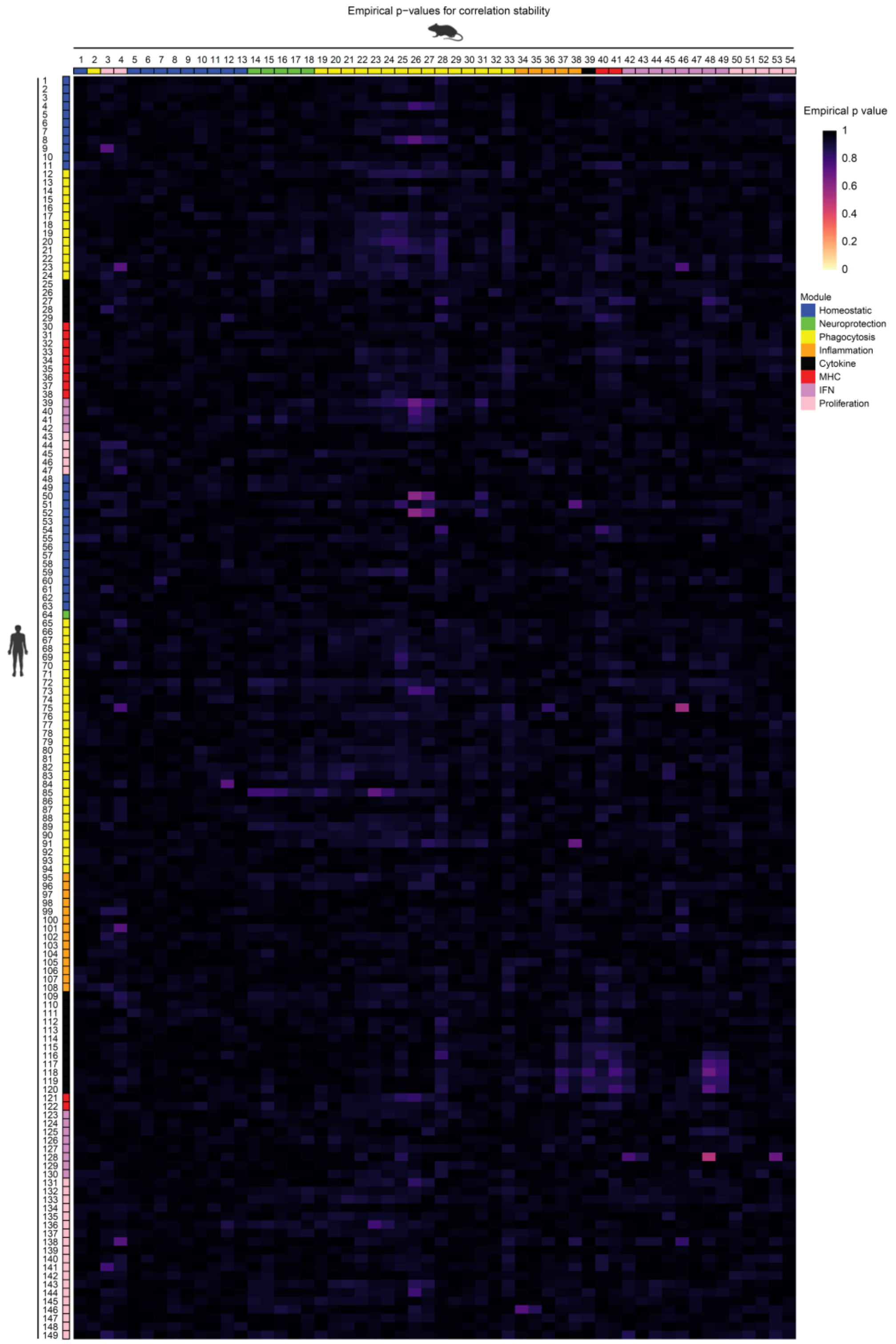

Supplementary Data Figure 2: Empirical *p*-values for correlation stability of microglia modules. Heat map showing empirical *p*-values for correlation stability of Human vs mouse microglia cluster gene expression shown in figure 6. Rows represent individual mouse microglia clusters, and columns represent individual human microglia clusters. Colors indicate empirical *p*-values (0 = unstable, 1 = stable).

Supplementary Table 1: Details of mouse models and time points of analysis.

Disease/Condition	Mouse model	CNS region	Time point of sample collection	Reference
Alzheimer's disease (AD)	5xFAD (FAD) model	Hippo-campus	16 and 40 weeks	[44]
	APP23 model	Hippocampus	1 year	[45]
Huntington's disease (HD)	B6CBA-Tg(HDexon1)62Gpb/3J JAX# 006494 R6/2 model	Striatum	15 weeks	[46,47]
Amyotrophic lateral sclerosis (ALS)	SOD1-tg (SOD) model B6.SOD1-G93A JAX# 004435	Cortex and spinal cord	15 weeks	[48]
Aicardi-Goutières syndrome (AGS)	Rnaset2$^{-/-}$ (Rnaset-2) model	Cortex	25 weeks	[49]
Viral encephalitis	Vesicular stomatitis virus (VSV) intranasal infection (i.n.)	Cortex and olfactory bulb	1 and 2 weeks	[50,51]
Multiple sclerosis (MS)	Cuprizone (Cup) de-myelination	Corpus callosum	5 weeks	[52]
	Cuprizone (Cup) re-myelination	Corpus callosum	1 week after 5 weeks of de-myelination	
	Experimental autoimmune encephalomyelitis (EAE)	Spinal cord Cerebellum Brain stem	Peak phase	[15]
		Spinal cord	Onset and chronic phase	
	Facial nerve axotomy (FNX) ipsilateral side	Facial nucleus	1 and 4 weeks	[53]
Sepsis	Intraperitoneal (i.p.) injection Lipopolysaccharide (LPS)	Cortex	24 hr	
Ischemic stroke	Middle Cerebral Artery Occlusion (MCAO) model	Cortex	1 and 2 weeks	[54]
Glioma	Glioma 261 model	Cortex	Endpoint	[55]
Familial Mediterranean fever (FMF)	FMF model (Mefv$^{V726A/V726A}$)	Cortex	32 weeks	[56,57]
B cell immunodeficiency	µMT mouse model B6.129S2-*Ighm*^{tm1Cgn}/J JAX #:002288	Cortex	24 weeks	[58]
Graft vs. host disease (GvHD)	CNS GVHD	Cortex	2 weeks	[59]
Embryonic microglia	E16 mouse brain	Whole brain	E16	[60,61]
Aging	Aged microglia (24 months)	Cortex	2 years	
Antibiotic treatment	Germ free (GF) model	Cortex	12 weeks and 2 years	[21,62]

Supplementary table 2: Antibodies used for imaging mass cytometry

Channel	Epitope	Manufacturer	Catalogue	Clone	Dilution
89 Y	CD45	Atlas	AMAb90518	CL0159	1:100
113 In	SPP1	Atlas	AMAb91653	CL10686	1:100
115 In	HLA-DR	abcam	ab176408	TAL 1B5	1:400
141 Pr	MBP	abcam	ab230378	EPR21188	1:100
142 Nd	Nestin	ProteinTech	29285-1-AP	polyclonal	1:100
143 Nd	GFAP	abcam	ab218309	EPR1034Y	1:200
144 Nd	ACSL1	ProteinTech	13989-1-AP	polyclonal	1:50
145 Nd	Olig2	R&D	AF2418	polyclonal	1:100
146 Nd	SOX2	Atlas	AMAb91307	CL4716	1:200
147 Sm	CD163	Novus	NB110-40686	EDHU-1	1:400
148 Nd	NeuN	Biolegend	834501	1B7	1:400
149 Sm	Vimentin	CST	46173SF	D21H3	1:200
150 Nd	CD56	Biolegend	318345	HCD56	1:200
151 Eu	CD31	Fluidigm	3151025D	EPR3094	1:200
152 Sm	Ki-67	Invitrogen	14-5698-82	SolA15	1:800
153 Eu	Neurofilament H	Biolegend	801701	SMI-32P	1:200
154 Sm	Map2	DAKO	M4403	HM-2	1:200
155 Gd	P2RY12	Atlas	HPA014518	polyclonal	1:800
156 Gd	CD4	Abcam	ab181724	EPR6855	1:200
157 Gd	FKBP5	Atlas	HPA031093	polyclonal	1:200
158 Gd	MX1	abcam	ab284604	EPR24485-19	1:400
159 Tb	CD68	Biolegend	916104	KP1	1:1600
160 Gd	HLA-DRA	ProteinTech	17221-1-AP	polyclonal	1:200
161 Dy	Iba1	abcam	ab220815	EPR16588	1:1600
162 Dy	CD8a	Biolegend	372902	C8/144B	1:800
163 Dy	TMEM119	Sigma	AMab91528	CL8714	1:800
164 Dy	XAF1	Atlas	HPA057302	polyclonal	1:400
165 Ho	HLA-DPA1	ProteinTech	16109-1-AP	polyclonal	1:800
166 Er	CD204	invitrogen	14-9054-82	J5HTR3	1:800
167 Er	CD11c	Proteintech	60258-1-Ig	2F1C10	1:200
169 Tm	SLC2A5	Atlas	HPA005449	polyclonal	1:800
170 Er	CD3	CST	85061S	D7A6E	1:800
171 Yb	SCAMP2	Atlas	HPA014699	polyclonal	1:800
172 Yb	Collagen IV	Millipore	AB769	polyclonal	1:1600
173 Yb	CD45RO	Fluidigm	3173016D	UCHL1	1:800
174 Yb	CD74	Biolegend	326802	LN2	1:200
175 Lu	CD34	abcam	ab198395	EP373Y	1:400
176 Yb	S100A9	Atlas	AMAb91690	CL11191	1:1600
194 Pt	HH3	CST	4499BF	D1H2	1:100
